# Recognising the deterioration of patients in acute care wards: a qualitative study

**DOI:** 10.12688/wellcomeopenres.17624.1

**Published:** 2022-04-19

**Authors:** Abi Beane, Wageesha Wijesiriwardana, Christopher Pell, N. P. Dullewe, J. A. Sujeewa, R. M. Dhanapala Rathnayake, Saroj Jayasinghe, Arjen M. Dondorp, Constance Schultsz, Rashan Haniffa

**Affiliations:** 1 Nat-Intensive Care Surveillance, MORU, Colombo, 08, Sri Lanka; 2 Mahidol Oxford Tropical Medicine Research Unit, Faculty of Tropical Medicine, Mahidol University, Bangkok, 10400, Thailand; 3 Nuffield Department of Clinical Medicine, University of Oxford, Oxford, OX3 7BN, UK; 4 Academic Medical Centre, University of Amsterdam, Amsterdam, 1105 AZ, The Netherlands; 5 Amsterdam Institute for Global Health and Development (AIGHD), Amsterdam, 105 BP, The Netherlands; 6 Monaragala District General Hospital, Monaragala, Sri Lanka; 7 Department of Medical Humanities, University of Colombo, Colombo, 8, Sri Lanka

**Keywords:** Recognition of deterioration, Critical care

## Abstract

**Background:** Infrastructure, equipment and staff constraints are often cited as barriers to the recognition and rescue of deteriorating patients in resource-limited settings. The impact of health-system organisation, decision-making and organisational culture on recognition of deterioration is however poorly understood. This study explores how health care providers recognise deterioration of patients in acute care in Sri Lanka.

**Methods:** In-depth interviews exploring decision making and care processes related to recognition of deterioration, were conducted with a purposive sample of 23 health care workers recruited from ten wards at a district hospital in Sri Lanka. Interviews were audio-recorded, transcribed and coded thematically, line-by-line, using a general inductive approach.

**Results:** A legacy of initial assessment on admission and inimical organisational culture undermined recognition of deteriorating patients in hospital. Informal triaging at the time of ward admission resulted in patients presenting with red-flag diagnoses and vital sign derangement requiring resuscitation being categorised as "bad". The legacy of this categorisation was a series of decision-making biases anchored in the initial assessment, which remained with the patient throughout their stay. Management for patients categorised as “bad” was prioritised by healthcare workers coupled with a sense of fatalism regarding adverse outcomes. Health care workers were reluctant to deviate from the original plan of care despite changes in patient condition (continuation bias). Organisational culture - vertical hierarchy, siloed working and a reluctance to accept responsibility- resulted in omissions which undermined recognition of deterioration. Fear of blame was a barrier to learning from adverse events.

**Conclusions:** The legacy of admission assessment and hospital organisational culture undermined recognition of deterioration. Opportunities for improving recognition of deterioration in this setting may include establishing formal triage and medical emergency teams to facilitate timely recognition and escalation.

## List of abbreviations

HCW: Health care worker

HIC: High income country

ICU: Intensive care unit

LMIC: Low- and middle-income country

SD: Standard deviation

## Introduction

Recognition of patient deterioration and timely response is a universal health care priority and a key component of high-quality healthcare ^1, 2^. In low- and middle-income countries (LMICs), where access to and availability of critical care services and complex interventions is limited, delays in recognition or escalation of deteriorating patients can be catastrophic for patients and families ^3– 5^.

In high-income countries (HICs), strategies to improve the recognition of deterioration have focused on investment in hospital-wide approaches to support clinical teams ^1^. These strategies have included assembling medical emergency teams, mandatory training for all frontline staff in recognising patient deterioration, and greater transparency in reporting adverse events and near misses to maximise learning from cases of failure to rescue ^6^. In addition, hospital-wide protocols for vital signs monitoring and early warning scores have become commonplace ^7^. Implementation of these strategies has required a shift in the organisation and processes of care for individual patients and a change in the behaviour of health care teams ^8– 10^.

In LMICs, infrastructure, equipment and staff constraints have been identified as barriers to the recognition of patient deterioration, with little attention given to the role of organisational structures, processes of care, or shared beliefs and practices among health staff ^11^. Poor understanding of these contextual factors hinders efforts to improve the quality of care and patient safety in LMICs ^12^. Drawing on interviews with healthcare providers in a district hospital in Sri Lanka, this article examines the factors that influence healthcare workers’ recognition of deterioration in ward patients.

## Methods

The study was performed from a critical realist perspective and adopted a general inductive approach to analysis ^13, 14^. This approach was selected given the need to explicate the complexities of the situational constructs, social hierarchies, organisational cultures and multiple interactions that likely influence health care workers' recognition of deteriorating patients ^13^.

### Setting

Interviews were conducted in a 370-bed district general hospital in Sri Lanka catering for an estimated population of 501,349 ^15^. Clinical facilities include 10 wards and an eight-bed intensive care unit. Supportive services include a medical laboratory, blood bank, haemodialysis services and radiology department. Staff at this hospital and all hospitals in the country are allocated to work in an institution by the Ministry of Health following a centralised system of training. Nurse to bed ratios on the wards range from 1:8 during the day to 1:14 at night ^16^.

### Respondents

Respondents were drawn from a purposive (diversity) sample of healthcare workers (HCWs), nurses, doctors and ward assistants/ attendants from all 10 wards. Quality improvement nurses (already working in the hospital setting) introduced the study team and approached HCWs to participate. All levels of staff seniority were invited to participate in an interview to enable maximum variation of perspectives ^17^. Recruitment continued until the point of theoretical saturation was reached i.e. when no additional information was forthcoming from subsequent interviews.

### Data collection

Interviews were conducted between December 2018 and February 2019. To increase the dependability of findings (reducing the impact of an individual researcher on data collection), interviews were conducted separately by two bilingual - Sinhala and English - research assistants with healthcare and qualitative research experience in similar settings. The interview approach, instructions for respondents, scene setting, participant-briefing and acquisition of informed consent were co-developed by the research assistants (WW and ND) and an experienced nurse researcher (AB) and incorporated into the interview guide ( *Extended data* ^18, 19^). Prior to collecting data, a series of training interviews were role-played to train the research participants, using practical examples of questions and answers designed to mimic potential respondent behaviours ^17^. A series of 5 pilot interviews were conducted with quality improvement nurses (all with significant clinical and contextual experience but who do not currently work in the clinical setting) to assess the interpretability and acceptability of interview questions and interview guide. These pilot interviews resulted in the addition of 2 opening questions (asked at the beginning of the interview) where the respondent was asked to recall and describe recent clinical events, during which they felt they had participated in recognition and management of a deteriorating patient. This opening question was designed to encourage the respondent to draw on real world experiences, recreating the situational conditions and encouraging respondents to recall interactions and communications prior to the subsequent interview question. The addition of this realist-rich data was intended to increase the validity and trustworthiness of findings ^20^.

Respondents selected a convenient time and place to be interviewed. All interviews were conducted in the hospital but away from clinical areas ensuring privacy and minimising interruption ^21^. Only the research assistants and the interviewee were present during the interview. Research assistants were not known to the respondents prior to the interviews. Interviews were conducted in Sinhala or English, depending on the healthcare workers preference and audio recorded.

Each interview began with a question to the interviewee to recall the last time they cared for a patient who went on to deteriorate in the ward. Further open-ended questions were posed to elaborate on the care that was subsequently provided and how the patient’s condition was monitored and deterioration identified (or not). Interviews focused on respondents’ experiences with specific patients to maximise their recall of what determined the care provided, maximising the validity of findings. More general questions allowed the researchers to assess whether these particular experiences reflected more general healthcare worker experiences, opinions and the interactions with other members of staff. The interview guide was iterated over the first five interviews in response to the initial analysis to elicit more detailed responses regarding the recognition of deterioration (Appendix 1 for English version interview guide).

### Data processing analysis

Audio-recordings were transcribed verbatim (WW and ND) into a text enabled e-data collection instrument in English within five days of the interview. Transcript names and content was standardised to facilitate analysis ^21, 17^. A random sample (n=8) ^17^ of the audio interviews was validated for accuracy of translation transcription by an independent member of the research team fluent in English and Sinhala (RH).

The data were analyzed using a general inductive approach, with analysis taking place alongside and after data collection. Transcriptions and coding were undertaken using Microsoft Excel (Microsoft Corporation. Microsoft Excel [Internet] 2018. Analysis was conducted by AB (a female health systems researcher and critical care clinician with more than 15 years’ experience in HICs and LMICs), WW and ND (allied healthcare professionals trained in Sri Lanka with qualitative research experience) supervised by CP (a social scientist with experience of health systems research in LMICs). Transcripts were coded line-by-line and emerging sub-themes were noted, compared, grouped and harmonised to identify core themes ^22^. During the course of the analysis, the core themes were used to develop a framework to explain how health care providers recognise deterioration in ward patients (Appendix 2). An iterative process of interview, transcription and analysis continued until data saturation, when no new themes were identified. The categories were then reviewed by the analysis team for consistency. To ensure transparency in the analysis process and increase the involvement of participants and end-users, themes arising from the interviews and the potential implications for future interventions to improve patient safety were fed back to the healthcare team as part of the ongoing collaboration.

### Ethical review

Permissions to conduct the study were obtained from the medical director and the matron of the hospital as part of an ongoing collaboration of quality improvement and research and from the Research and Ethics Committee. An information leaflet was given to each prospective interviewee detailing the aims of the research and its relevance to quality improvement 48 hours ahead of the interview (Additional file 2). Interviewees were reassured that they could pause, stop or withdraw from the interview at any time during or after the interview with no implication on participation in future work. Interviews and analysis were carried out in accordance with the Declaration of Helsinki and the study has been approved by the Ethics Review Committee of the Faculty of Medicine, University of Colombo, Sri Lanka (EC-15-034, version 3). Oral informed consent was taken at the beginning of the interview; the participant information statement (including ability to withdraw) was read to the interviewee, and then their decision to consent was recorded on the audiofile and anonymity was assured. The IRB confirmed oral consent was adequate for this study as the interviews were audio recorded. Audio Files were downloaded and saved as part of study documentation.

## Results

**Figure 1.**
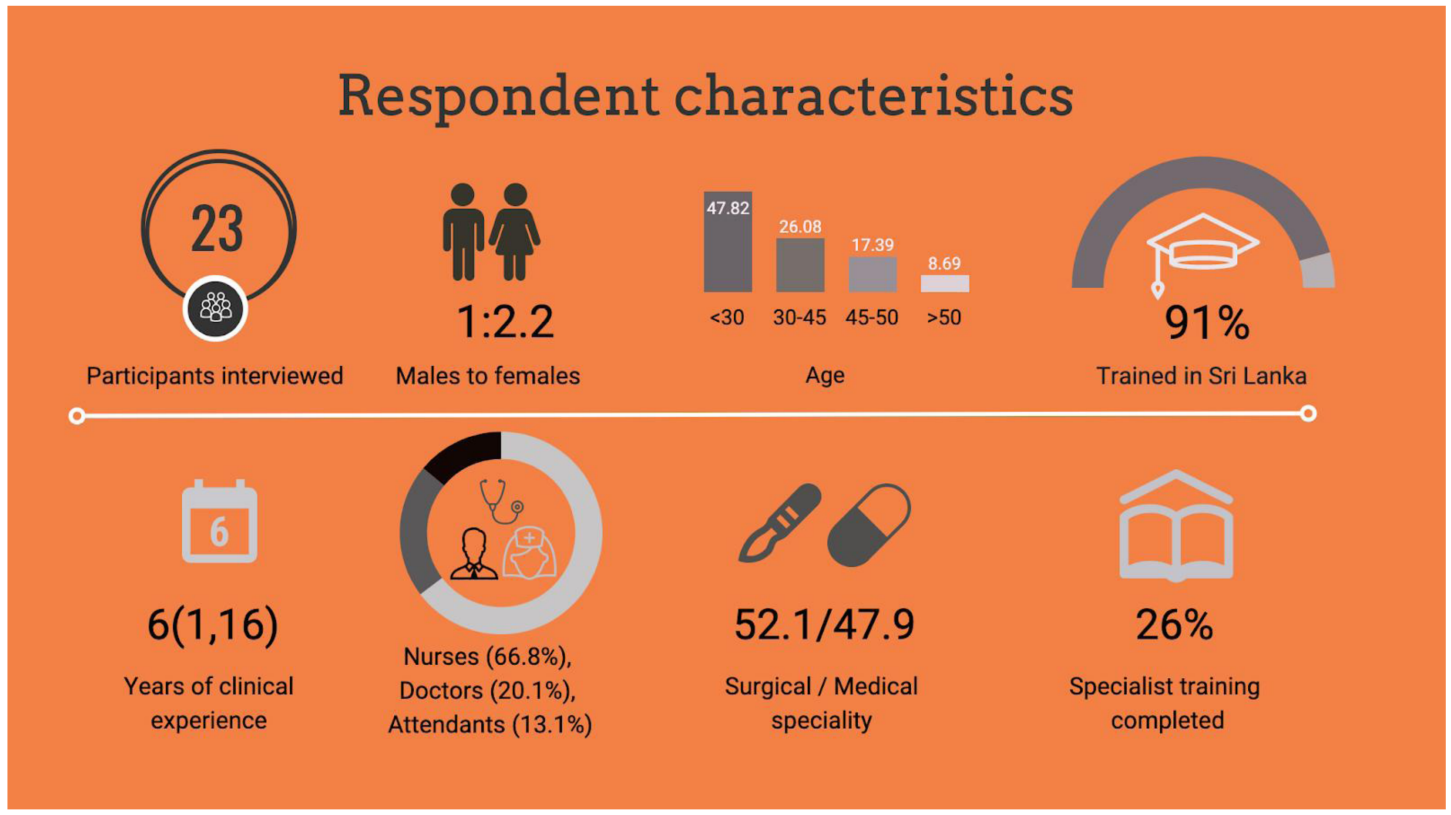
Respondent characteristics.

**Figure 2.**
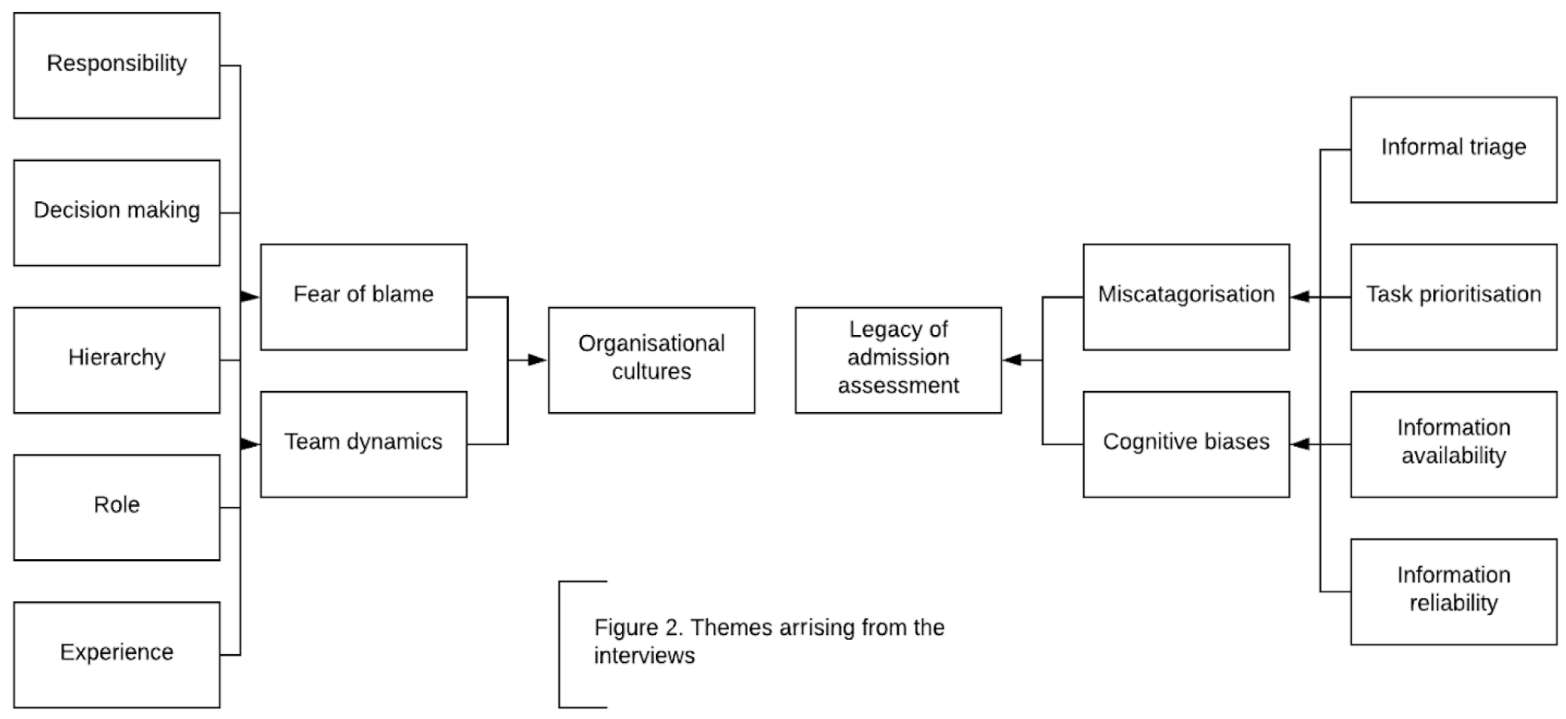
Emerging themes.

In total, 24 healthcare workers were invited to participate in interviews. Respondent characteristics, training and experience is described in Figure 1. Respondents were drawn from all cadres of frontline healthcare workers that have direct contact with patients during their journey through acute care. One healthcare worker declined participation because of concern over how senior staff may perceive the study findings. Overall, 23 respondents provided 22.4 hours of interview data ^23^. On average, an interview lasted 44 minutes (standard deviation [SD] 22.4 minutes). Two core themes emerged from the analysis; legacy of ‘admission assessment’ and ‘organisational cultures’, (which included the sub themes of ‘team dynamics’ and ‘fear of blame’) ( Figure 2).

### Legacy of admission assessment

On admission to the ward, junior doctors undertook an informal triage of patients. This triage was pivotal to the ward team’s recognition of subsequent deterioration. Although no explicit triage tools were in use, based on patients’ clinical presentation and working diagnosis, junior doctors characterised a minority of these patients as “bad”. The diagnoses perceived as high-risk included: snake bite, road traffic injuries, stroke, leptospirosis and fever.

*So, we know from the admission. First, when we receive a patient, we take the admission sheet and, if it is an [emergency] case, we inform the doctor, then we take the patient to a bed. After taking the patient to a bed we check the patient and inform the doctor to assess. We observe pulse, respiration and body weight. [When we have a diagnosis] we can prioritise the patients better when we know what’s wrong.*
  Nurse, surgical ward

*[B]ad patients… I have experience. After working with patients, we know after some point the patient will get bad. I get that idea from seeing and observing a lot of patients arrive with the same thing – fever, headache, they get drowsy...*
  Nurse, medical ward

Similarly, patients recognised as acutely unwell at ward presentation and requiring interventions, such as oxygen, fluids, intravenous antibiotics or imaging were also labelled as “bad”. Conversely, absence of a diagnosis that junior doctors recognised as “high risk” or physiological instability requiring resuscitation on admission meant patients were not triaged as "bad". Patients who were not characterised by healthcare workers as “bad” included those presenting with chronic conditions, such as ischemic heart disease, chronic renal disease and those presenting for routine investigation and planned surgery. Individuals presenting with symptoms, such as chest pain that was not obviously attributable to cardiac causes, abdominal pain not requiring immediate interventions, nausea, vomiting or diarrhoea were unlikely to be identified as "bad".

*It is hard to tell MI patients from gastritis patients; since they both complain of chest pains – it’s difficult to tell. Chest pain, sweating, vomiting as general presentation. These are the difficult ones. There may be more types of chest pains. [For example,] chest pain due to digestive problem. Only when the pain is radiating through the arm, then we know it is more critical…. Sometimes they come in complaining but then they settle quickly. I usually leave them for a bit when they first come in. [Although] sometimes they want attention. Especially if the doctor doesn't seem concerned. Give them some time. They get anxious and then they feel they are worse than they are… *
  Nurse, medical ward

Although aware that these symptoms may be part of serious underlying conditions, respondents described how doctors were often reluctant to label such patients as "bad". Patients previously admitted to the same ward or who were known to the team were less likely to be assigned the label of "bad".

Healthcare workers described how patients who have been labelled "bad" on admission experience a higher risk of subsequent deterioration. This notion resulted in a higher frequency of vital sign monitoring, allocation of a bed closer to the nursing station, family members or attendants being encouraged to stay, and a greater priority being placed on obtaining and reviewing laboratory information and imaging in support of the working diagnosis. In contrast, those not identified as "bad" on admission were seen by health care workers as at lower risk of deterioration during the immediate period following admission and throughout their hospital stay.

*Once we know the patient is bad, we monitor them check [their vitals, and their bloods] and observe regularly. […] We get criticised if not.*
  Nurse, medical ward

Actions that might assist staff to detect deterioration, such as locating patients close to the nursing station, increasing the frequency of vital sign recording, reassessment or re-categorisation, were given a lower priority. Although respondents acknowledged that the absence of vital signs was a barrier to recognising deterioration for all patients, the failure to measure and record vital signs for patients who were without the characterisation of "bad", was seen as unavoidable.

Healthcare workers expressed a sense of inevitability (‘fatalism’) in the event of adverse events, such as intensive care unit (ICU) admission, cardiac arrest or death for those patients categorised as "bad". Whereas healthcare workers perceived patients with this characterisation as having a greater risk of deterioration, treatment goals, trajectory of illness and prognosis were not discussed formally within the multidisciplinary team, or with patients or family members as part of care planning.

*I was really surprised he got bad […] he was OK for the first three after surgery. Only then he got bad. Since the patient was good for the first two days after surgery we thought that he would be OK. He had a fast pulse, but I thought like it was the fever. I thought it would settle. […The] patient was getting worse during the last 2 days but the arrest happened suddenly… [death] wasn’t expected. I was really surprised. But it was OK. [I] guess it was just going to be like that.*
  Junior doctor, medical ward

Although respondents also described inevitability about the outcomes of patients triaged as “bad”, they were surprised when patients not triaged as "bad" went on to experience adverse events. Deterioration in these patients was perceived to be unpredictable and unpreventable- even with antecedents, such as derangement in vital signs or commencement of antibiotics.

*I did not expect him to die, he was not bad when he came to the ward. I remember – he has come before and he looked the same. He was well when he arrived… He always comes [for his dialysis]. At the beginning he was in the chair, then later bedridden. He wasn’t like he was before.*
  Nurse, medical ward

Deranged vital signs did not prompt a re-categorisation of patients to “bad” or a prioritisation of subsequent vital signs monitoring. Providing emergency care and resuscitation for patients with characteristics of acute illness at admission were rather seen as a higher priority than re-assessing established ward patients. Moreover, nurses, in particular, described how they could not predict deleterious outcomes for patients, regardless of their status at admission.

### Organisational culture

The theme organisational culture relates to the discrete and tacit shared behaviours, practices and beliefs within the hospital ^9^.

***Team dynamics.*** A strong vertical hierarchy was evident within the ward team, with all healthcare workers identifying the consultant (attending physician or surgeon) at the apex. Ward rounds were described as almost exclusively consultant-led with consultants determining the sequence of the ward round, the time of round, prioritisation of patients for review, and major decisions regarding the direction of care and interventions.

*I lead my team and make the decisions regarding care. I then explain to the juniors…...they can ask me anything...ward round is for teaching them. I have set that example on my wards.*
  Lead senior clinician, medical ward

All responded expressed deference towards their superiors when describing care practices and decision making. An absence of a senior doctor both on a ward round and during individual patient reassessment was perceived to be a barrier to recognising a deteriorating patient.

*[W]hen [the] patient is deteriorating, the patient is identified, then we inform the doctor. After that nurses help the doctor according to the doctor's decisions about the plan. Sometimes we wait for the consultant – but they usually come quick – then we can manage...*
  Doctor, surgical ward

Doctors and nurses described separate roles in detecting deterioration. A junior doctor’s role included formal ward-based assessment and reassessment. Consultants and senior clinicians, who had responsibilities outside the ward in clinic and theatres, would review patients at ward round or in response to an alert from a ward-based doctor. In contrast, nursing roles were task focused.

*Doctor tells [us] what to do. The doctor is saying the frequency [of vital signs measurement] and then we follow. Sometimes it’s a lot... doctors assess and identify the patients as bad, then we [the nurses] can monitor them…...minor staff and family, they help. […] When we come to the wards, we check the patient for the ward round, handover and then the doctor can assess. It’s important that we have the charts ready for the ward round. Sometimes it’s a rush to finish for the round, but they are important then. Through the ward round they can assess the information and [the doctors] decide.*
  Nurse, surgical ward

These separate roles, along with an individual's position within the team’s hierarchy, influenced respondents’ sense of empowerment to recognise deterioration in patients, and in their perception of responsibility for missed recognition. Doctors perceived hierarchy to be positive, empowering them to participate in collective decision-making. For nurses, responsibility was perceived negatively and was strongly linked with concerns over blame and personal criticism.

*“[S]ometimes the relatives tell us we have not paid attention to their patient, the relatives are criticising, they are saying like [the] patient was good in the morning, but now at lunch time the patient is bad. The patient's relatives will criticise that the nurse didn't pay attention to the patient. It makes me feel bad. It’s different if we can get the doctors to speak with them. Once the doctor explained then it was ok..”*
  Nurse, medical ward

The differing roles and perceptions of responsibility within the team resulted in varied approaches to decision-making. Junior doctors’ decisions to categorise a patient as “bad” at admission was primed by experience and recognition of patterns of illness associated with “high risk” diagnosis. Their subsequent decisions regarding patient care were approached collaboratively, but within their peer group. They cited opportunities to discuss clinical findings with senior doctors as a key component of their ability to recognise deterioration. Characteristics of collective decision-making included face-to-face discussion at ward round and remotely via messaging applications or telephone calls.

*My current consultant – he is supportive if we make decisions, and he helps us [the doctors] plan ahead. Then we know what we can do and what not. My colleagues and I text each other. That helps, especially at night. It feels better to check with someone that way, otherwise it can be a long time to wait till the morning.*
  Junior doctor, surgical ward

On the other hand, senior doctors and consultants described more individualistic styles of decision-making. Not restrained by hierarchy, their decision-making style signified a strong sense of self-belief, whereby experience and an ability to make rapid decisions under pressure were valued highly. Furthermore, they saw themselves as having overall responsibility for patient care.

In contrast to doctors, nurses recounted only limited involvement in decision-making and were absent from collective decisions. Although present during ward rounds, nurses were reluctant to contribute to the collective discussion regarding patients’ progress or treatment goals. Uncertainty over treatment goals and prognosis resulted in inertia in nursing practice, particularly in terms of the re-categorisation of patients identified as "bad" at admission when faced with signs of improvement. Similarly, they expressed reluctance to escalate the frequency of vital signs in patients not identified as "bad" on admission, even if they deteriorated.

***Fear of blame.*** Fear of blame inhibited nurses’ and junior doctors’ ability to re-prioritise tasks that could enable timely recognition of deterioration. Although healthcare workers of all cadres were concerned about blame for not completing vital signs and for failure to recognise deterioration, this was most evident amongst nurses. In comparison to doctors, who recounted how events including failure to rescue provided them with opportunities to learn, nurses associated such events with blame, recalling how they would be personally criticised if information which could be perceived to enable recognition of deterioration in "bad" patients was missed.

*If I miss a dengue patient getting bad there will be criticism from the other team members. We know these patients are risky, especially in the first few days. I feel sad if I miss them getting bad...[…] It’s not all about criticism; it’s a kind of lesson not to do that again. Seniors are showing what are the steps missed and not done properly. […]*
  Junior doctor, medical ward

Fear of blame was the catalyst for monitoring "bad" patients. Doctors and nurses viewed with suspicion information that was contradictory to the initial categorisation of patients and recalled how they would often repeat vital signs and assessment in the event of deterioration.

*I will want to check [vital signs] for myself once a nurse or junior tells me a patient is bad. I need to be certain. I will be the one speaking to the consultant and I need to be sure of my facts before I call him...*
  Doctor, medical ward

*I always feel more anxious when I am working with junior nurses. She might not know what to look for or might not record correctly. Then I will have to check myself… If I don’t check myself then it will be me who is blamed.*
  Senior nurse, medical ward

This misgiving was heightened if the source of information was a team member perceived to be junior. Junior doctors and nurses recounted how they would sometimes defer decision-making until seniors were present at review to further avoid criticism. The fear of blame extended to the three-way relationship between healthcare workers, patients and any bystanders. Junior nurses, in particular, were concerned that family members would be critical in the event that their relatives deteriorated.

*Even if we [the nurses] miss a little thing about the patient, [the relatives] will complain about it to us. They do not feel we have cared until we do something. So that is why we need to focus and build trust among the patients, not only the bad patients: the normal patients also. Otherwise it's tough on us…. I feel proud when we recognise the patient is bad. Then we can send them home- they get better. The family are always so grateful. It’s a blessing.*
  Senior nurse, surgical ward

## Discussion

The legacy of the assessment at the time of ward admission is an alteration of the healthcare worker heuristic when encountering the deteriorating patient. The informal triaging of patients presenting with red-flag diagnosis or vital sign derangement requiring immediate intervention as “bad”, inhibits healthcare workers from recognising deterioration early in other patients and leads to resistance to seek out and act on information that might challenge the initial categorisation ^24^. Limited opportunities for collective goal-setting and interdisciplinary discussions regarding prognosis, contributes to a sense of healthcare worker (HCW) fatalism regarding deterioration in patients identified as "bad" on admission. As described elsewhere, strong vertical hierarchy, fear of blame, fragmented roles and negative perceptions of responsibility contribute to delays in recognising deteriorating patients and hinder future improvement initiatives ^8, 25^.

The informal triage system leads to inconsistencies and mis-categorisation due to inter and intra-observer variability and unintended consequences ^26, 27^. Excessive dependence on diagnostic red flags by HCWs may result in mis-categorisation due to misdiagnosis and atypical presentation at admission ^16, 25, 28^. For example, during the annual dengue season, triaging based purely on empirical clinical diagnosis can lead to the number of "bad" patients (and workload) increasing manifold, overwhelming healthcare workers and impeding quality of reassessment. This overreliance on the initial assessment (anchoring), resulted in a series of cognitive biases in healthcare workers’ subsequent decision-making ^24^.

The legacy of this informal triage system is linked to the misperception that patients not triaged as “bad” at admission are at virtually no risk of subsequent deterioration. This bias was evident in healthcare workers’ reluctance to deviate from this initial risk assessment even when there was evidence of subsequent deterioration in the clinical status in patients ^24, 28, 29^. The consequences of anchoring decisions in the initial assessment include limited vital sign recording in patients not identified as “bad” (confirmation bias) and a reluctance to re-catagorise patient risk, even in the presence of antecedents to adverse events (known as plan continuation bias) ^30^. The absence of reliable, regular and complete vital signs inhibits the identification of patients whose deterioration may be preventable and any opportunity to learn from cases of failure to rescue through mortality reviews and activities designed to enable reflection and learning, such as an After-Action Review ^25^.

In many contexts, the fear of blame is a strong impediment for healthcare systems to learn from failure to rescue deteriorating patients ^31, 32^. Fear of blame and inimical organisational behaviours hinder individual healthcare worker and teams self-reflection about processes of care and the consequences of decision-making ^8, 33, 34^. In this study, concern over blame from peers and the wider public deterred nurses and junior doctors from revising the initial categorisation, and is a driver for the consistent prioritisation of vital signs in patients labelled as "bad". The fear of blame, rather than fear of failure to rescue, motivated vital sign recording. 

Healthcare workers’ reluctance to take responsibility for decisions (omission bias), results in their failure to respond to changes in patients’ condition – a phenomenon described in HIC settings and in other industries seeking to improve safety in Sri Lanka ^24, 35^. Similarly, institutionalised vertical hierarchies, as observed in this study, have been identified as impediments to delivery of high-quality care and a barrier to improvement ^9, 25, 34, 36^. Opportunities for improving recognition of deterioration in this setting, require a shift in organisational culture away from blame or criticism of individuals and towards a shared institutional responsibility ^9, 37^.

Implementing an explicit triage system for patients on ward admission could improve the recognition of unwell patients that require immediate intervention and the stratification of those at increased risk of subsequent deterioration ^27, 30^. The risk stratification can help explicitly guide both the frequency of nurse recorded vital signs and junior doctors’ reassessment. Incorporating reassessment into the ongoing care pathway may help provide information to challenge the initial assessment and the cognitive biases which currently influence subsequent decision making.

By diverting immediate resuscitation away from the busy, under-resourced and unprepared ward to emergency units, theatres and intensive care where senior staff with specialist training and higher nurse to patient ratios are available, minimises the conflicting priorities for ward teams between immediate resuscitation and care of established patients. The ongoing restructuring of health facilities to incorporate formal emergency departments in the region provide an opportunity to improve organisation of care ^38^.

In many HICs, multidisciplinary medical emergency teams provide support for junior ward staff in assessing, managing and transferring acutely unwell patients. In this and other LMIC health systems, where inexperienced ward teams are often at the frontline of assessing and identifying patients who may be at risk of deteriorating, such rescue teams could be hugely beneficial ^39^. In addition, they could provide a conduit of communication between junior and senior ward staff and critical care teams, facilitating ICU admission, and when appropriate, discussions regarding prognosis and end of life care.

### Strengths and limitations

The strengths of the study included: interviewing a diversity of healthcare providers; using an in-depth interview guide that focused on specific events and general experiences/opinion; involving a team of researchers with varying experience and backgrounds (all with training in the social sciences); two researchers separately conducting interviews; checking of transcription, translation and coding during the analysis process by an additional researcher, reporting the emerging findings from thematic analysis back to the respondents and the potential end-users. The findings are limited by the single data collection setting: one LMIC hospital. Nonetheless, the study illustrates how organisational cultures and processes of care may influence recognition of deterioration, which are likely to be relevant in acute care settings elsewhere in South Asia and beyond. The extent to which these findings reflect the situation in other hospitals across the region and in other LMICs is the subject of ongoing work undertaken by this group.

## Conclusion

The legacy of informal triage at ward admission by junior doctors and an inimical organisational culture- characterised by vertical hierarchy, task-based role separation between doctors and nurses and an overarching fear of blame, undermined recognition of deterioration. Opportunities for improving recognition of deterioration in this setting - and likely elsewhere across the region and in LMICs - include establishing formal triage systems, implementing medical emergency teams to support ward based HCW and facilitating a shift in organisational culture including through opportunities to learn from failure to rescue.

## Data availability

### Underlying data

Figshare: Interview transcripts and analysis. https://doi.org/10.6084/m9.figshare.19207215.v3 ^23^.

### Extended data

Figshare: Additional File 1: Interview guide. https://doi.org/10.6084/m9.figshare.19102370 ^18^.

Figshare: Additional File 2: Information sheet/leaflet. https://doi.org/10.6084/m9.figshare.19102388.v1 ^19^.

Data are available under the terms of the Creative Commons Zero "No rights reserved" data waiver (CC0 1.0 Public domain dedication).
