# Supplementary material for: Recognising the deterioration of patients in acute care wards: a qualitative study
Source: Wellcome Open Res. 2022 Jun 13;7:137. Originally published 2022 Apr 19. [Version 2] doi: 10.12688/wellcomeopenres.17624.2 (PMC10435917; doi:10.12688/wellcomeopenres.17624.2)
Supplement: Peer review history

# Peer review: Reviewer response for version 2

**DOI:** 10.21956/wellcomeopenres.19906.r62562

**Published:** 2023-08-17

**Authors:** John Rihari-Thomas

**Affiliations:** 1 University of Wollongong, Wollongong, Australia; 2 St Vincent's Health Network, Sydney, Australia

This paper discusses similar barriers to existing international bodies of knowledge around late detection of patient deterioration. Where it does differ is in explaining the cultures and hierarchies within a Sri Lankan context. For this reason it is an interesting read and good themes emerged from data analysis giving insight to Sri Lankan clinician 'ways of working'. 

 In summary, it demonstrates barriers to identification and escalation similar to other literature, not surprising as this phenomena is a global issue. The limitation was acknowledged by the authors that this is a single site study, if the journal is happy with this I am supportive of indexing given it's distinct look into the Sri Lankan deteriorating patient setting.

Is the work clearly and accurately presented and does it cite the current literature?

Yes

If applicable, is the statistical analysis and its interpretation appropriate?

Not applicable

Are all the source data underlying the results available to ensure full reproducibility?

Yes

Is the study design appropriate and is the work technically sound?

Yes

Are the conclusions drawn adequately supported by the results?

Yes

Are sufficient details of methods and analysis provided to allow replication by others?

Yes

Reviewer Expertise:

Cardiology, patient deterioration, medical emergency teams, patient safety.

I confirm that I have read this submission and believe that I have an appropriate level of expertise to confirm that it is of an acceptable scientific standard.

# Peer review: Reviewer response for version 2

**DOI:** 10.21956/wellcomeopenres.19906.r62565

**Published:** 2023-08-17

**Authors:** Tracy Flenady

**Affiliations:** 1 Central Queensland University, Brisbane City, Australia

**Introduction:**

 References for the opening paragraph are up to 14 years old. In particular, the reference used to make the point about strategies to improve the recognition of deterioration, which is a major rationale for conducting the study  (1 ^st^ sentence second paragraph in the introduction) is outdated and redundant.

**Methods:**

- Where is your research question? Research aim?
- How many interviews? (As I read on, I see this is listed in the results section. This is too far into the manuscript and should be included earlier).
- Over what period of time were the interviews conducted?
- Number of interviews and demographics of respondents should be included under the heading ‘population' or ‘respondents’.

**Results:**

- There is methodological information in the results section. Only results should be listed in the results section.
- You need a definition of ‘bad’ early on in the results. I don’t know what ‘bad’ means in terms of your study.

**Overall:**

- Please include a definition of 'bad' in the context of your study.
- Please increase the information in the methods section.
- Remove methodology information from the results section.
- The references need updating.
- I don’t know what the research question is, so I don’t know if the methodological approach was suitable. Please make this clearer.
- I don’t know if the researchers addressed their study’s aim because it was not listed. Please make this clearer.

Is the work clearly and accurately presented and does it cite the current literature?

Partly

If applicable, is the statistical analysis and its interpretation appropriate?

Not applicable

Are all the source data underlying the results available to ensure full reproducibility?

Yes

Is the study design appropriate and is the work technically sound?

Yes

Are the conclusions drawn adequately supported by the results?

No

Are sufficient details of methods and analysis provided to allow replication by others?

Partly

Reviewer Expertise:

qualitative methods. detection of clinical deterioration, specifically, early warning systems.

I confirm that I have read this submission and believe that I have an appropriate level of expertise to state that I do not consider it to be of an acceptable scientific standard, for reasons outlined above.

# Peer review: Reviewer response for version 2

**DOI:** 10.21956/wellcomeopenres.19906.r51032

**Published:** 2022-06-14

**Authors:** Kate Grailey

**Affiliations:** 1 Department of Surgery and Cancer, Imperial College London, London, UK

Thank you to the authors for addressing the points raised within my review - this updated manuscript reads well and with increased clarity. 

 This is a valuable piece of work that will be of interest to all those working within the healthcare profession, particularly within the acute care setting.

Is the work clearly and accurately presented and does it cite the current literature?

Yes

If applicable, is the statistical analysis and its interpretation appropriate?

Not applicable

Are all the source data underlying the results available to ensure full reproducibility?

Yes

Is the study design appropriate and is the work technically sound?

Yes

Are the conclusions drawn adequately supported by the results?

Yes

Are sufficient details of methods and analysis provided to allow replication by others?

Partly

Reviewer Expertise:

Qualitative research, organisational psychological

I confirm that I have read this submission and believe that I have an appropriate level of expertise to confirm that it is of an acceptable scientific standard.

# Peer review: Reviewer response for version 1

**DOI:** 10.21956/wellcomeopenres.19496.r50388

**Published:** 2022-05-10

**Authors:** Kate Grailey

**Affiliations:** 1 Department of Surgery and Cancer, Imperial College London, London, UK

This is a very interesting and well written paper which I enjoyed reading. Minor suggestions only, particularly including some clarity around the qualitative analysis technique. This is an under-researched concept, potentially with huge implications for patient care. As such, this manuscript is a valuable addition to the literature.

 Abstract:

- Consider changing the word “bad” in the results – perhaps “unwell” might be a more appropriate word?

Introduction:

- You could consider adding in some data to support the strategies used by HIC to recognise the deteriorating patient.

Methods:

- Within the data collection section, you state that “a series of training interviews were role-played to train the research participants”. I assume you mean to train the research assistants who were to be conducting the interview, but clarity could be improved here to avoid misunderstanding.
- Consider expanding on and clarifying your qualitative analysis technique – you describe a thematic analysis approach, with a framework being constructed inductively & led by the data.
- It would be beneficial to include a short paragraph on reflexivity within the methods section.

Results:

- You describe using an assessment of thematic saturation in the methods – was this achieved?
- You describe reviewing themes within the team for consistency – were there any discrepancies?
- Again, use of the word “bad” seems a little out of place in the context of an unwell patient. Consider changing this to “unwell” or “high risk”. Or if “bad” was decided upon based upon the content of the interviews and language used by individuals, this should probably be explained within the text. The word “bad” feels quite distracting when reading the manuscript.
- Typo – “all responded expressed deference” – suggest changing to respondents.
- Consider clarifying seniority of participants in the descriptors after the quotations – some are just listed as “doctor” or “nurse”.
- Consider explaining who you are referring to with the term “bystanders” – members of the public? Patients?

Discussion:

- Suggest including other study limitations, such as bias associated with purposive sampling, generalisability, methodology.
- Have any similar studies been conducted where initial triage and assessment affect subsequent recognition of deterioration?

Is the work clearly and accurately presented and does it cite the current literature?

Yes

If applicable, is the statistical analysis and its interpretation appropriate?

Not applicable

Are all the source data underlying the results available to ensure full reproducibility?

Yes

Is the study design appropriate and is the work technically sound?

Yes

Are the conclusions drawn adequately supported by the results?

Yes

Are sufficient details of methods and analysis provided to allow replication by others?

Partly

Reviewer Expertise:

Qualitative research, organisational psychological

I confirm that I have read this submission and believe that I have an appropriate level of expertise to confirm that it is of an acceptable scientific standard, however I have significant reservations, as outlined above.

# Response

**Published:** 2022-06-02

**Authors:** abi Beane

**Affiliations:** Nuffield Department of Clinical Medicine, Oxford., UK

The authors thank reviewer [1] for the positive peer review and have considered the recommendations to improve the manuscript. These recommendations have been included in this revised submission. We hope these revisions address the reviewers recommendations, and provide greater clarity to the reader.
